# Protecting the Nation from Health Security Threats

**DOI:** 10.1089/hs.2016.0122

**Published:** 2017-02-01

**Authors:** Tom Inglesby, Anita Cicero

Events in the United States and internationally have shown the kinds of great stakes and consequences that often follow epidemics and disasters. Consider the series of health security shocks since 2001: National fear during the anthrax letters events, with impact on all 3 branches of the US government. The respiratory transmissible SARS and MERS viruses spreading via airplanes and hospitals, sometimes by super-spreading individual patients. Human cases of H5N1 bird flu with a case fatality rate of 50%. The sudden emergence and global spread of 2009 H1N1. The congenital malformations following Zika infections—the first mosquito-borne illness to cause such malformations. The terrible mortality and wide spread of Ebola across multiple countries in West Africa, with imposition of large-scale quarantines and costly economic disruptions to trade and travel. The widespread disabling of the healthcare system during major US hurricanes. The use of chemical weapons in war in the Middle East, breaking years of international taboo against it. Discovery of smallpox in an insecure box in a US government lab. The radiation risks and uncertainties over large areas following the Fukushima tsunami and nuclear power plant accident. High-containment laboratory accidents involving important live pathogens.

Beyond these events, there are other important potential threats that could cause great harm and test our systems of health preparedness in the United States and internationally, including large earthquakes, substantial terrorist attacks, and nation state or terrorist use of biological, chemical, radiological, or nuclear weapons—events that could have the potential for great loss of life. In the realm of synthetic biology, the possibility of generating novel laboratory strains that have both high lethality and are readily transmissible is an important new challenge that the world needs to manage.

These are problems that have the potential to cause sickness, mortality, and disruption on a large scale. But the consequences are not limited to health. Outbreaks and other disasters can come with the potential to have an impact on economies by shutting down commerce, or by causing widespread anxiety that affects the public in ways that have ripple effects on work and spending. Some outbreaks have caused governments to make bad choices to quarantine people in ways that did not end epidemics but that did cause loss of trust in government. Some of these events have reduced confidence in and credibility of political leaders.

***Health security*** is the collective effort to prevent, mitigate, and recover from the health consequences of these kinds of epidemics, disasters, and catastrophes. In many of these events, major initial impacts are related to health and loss of life. But when health consequences of this nature and potential scale occur, or are legitimately feared, then economic, governance, political, and security consequences can quickly follow.

The US government—working with partners at the state and local levels, the NGO community, the private sector, and the public—has done an enormous amount in the past 15 years to prepare for these kinds of events at home and, to a lesser extent, to help build strength abroad in ways that protect other countries and our own. But even with all this work, it's clear that our preparedness for these kinds of problems is often not substantial enough for the big challenges we face. In many cases, US programs are too small, the funding is too jerry-rigged, or we haven't made the necessary investment in technology or in people.

In the past, prominent government leaders have asked, “Isn't the work of preparing for large-scale health emergencies completed yet?” The answer is that health security is not something that gets completed. Just like national security is not something that is ever fully completed. Long-standing threats continue to persist and new challenges emerge, so it is crucial to remain prepared and keep looking ahead. Talent must be recruited, and new systems have to be built and evolve. And if we don't continue to support these efforts, the infrastructure behind them will degrade and preparedness will suffer.

Even for the systems that have been built and are strong, there are limits. The United States has not confronted truly large-scale catastrophes, as might for example happen with a novel pandemic disease. It has been hard enough to build systems that address modest-sized epidemics and disasters.

One of the primary functions of government is to protect its people from the greatest dangers they might face. Larger-scale biological catastrophes should be on that list of potential greatest dangers, so we need some aspect of our national preparedness effort to be working to diminish the likelihood of those kinds of events and to lessen the consequences of potentially globally stunning events. As part of that work, we need a better understanding of the pathogens and conditions that could cause new biological catastrophes. At a basic level, we also need a way of communicating and planning around these events, with language that is clear and doesn't lead to people becoming resigned to suffering the consequences of these events or dismissing the possibility of their happening.

The transition to a new Administration and Congress is a critical time to take stock. It is a time to make adjustments and consider ways of making programs more effective and efficient. But most important, the beginning of a new administration presents a unique opportunity to institutionally and politically emphasize new (or as yet unachieved) goals that will set the direction for programs and agencies.

Our colleagues at the Center for Health Security have written a series of commentaries that follow this one that provide facts and assessments regarding what has been established in key realms of health security and what needs to be done now. We highlight some of these recommendations below and give our thoughts on ambitious goals that could, if embraced by the new Administration, significantly advance our national ability to save lives, economies, and societies when faced with serious health security threats. Some of these goals have been aspired to for a long time, but there has not been the kind of national commitment to fully achieve them. The new Administration and Congress could collectively realize all or part of this vision and transform health security in the United States.

➢ **The United States needs to improve the ability to develop new medical therapies and vaccines to cope with biological threats the country may face.**

The United States should have, but currently does not have, the capacity to develop a new vaccine or medicine quickly for any novel biological threat that we face from either nature or a deliberate attack. Our nation has invested in and built impressive systems for research and development of medical countermeasures for biological threats, antimicrobial resistance, pandemic flu, and emerging epidemics. It has a program that draws on the talents and science of NIH, ASPR/BARDA, FDA, CDC, and DoD (also known as the Public Health Emergency Medical Countermeasures Enterprise, or PHEMCE), working closely with biopharma companies. The United States and other countries have depended on our systems during past events. For example, the US government and its biopharma partners led a global development effort for medicines and vaccines after anthrax 2001, for H5N1 bird flu, for 2009 H1N1, for Ebola, and now for Zika, as only a partial list.

Even with these successes, the drug and vaccine development process has mostly taken too long to make a difference during the most important period of a new outbreak. For instance, it is likely to take at least 6 months after the discovery of a new flu pandemic for this system to produce an effective vaccine. And flu is the best case. For many other new threats, it can take a decade or longer to develop new medicines and vaccines. We agree with the recent President's Council of Advisors on Science and Technology report, which called for the US government to develop the national capacity to make a new vaccine or medicine for any new biological threat within 6 months. To get there, this goal must be made explicit, technology investments will be required, and progress toward it should be tracked.

In the meantime, as the government works toward that longer-term goal, the programs of government that develop medical countermeasures in partnership with industry need to be supported and strengthened, and talented people need to be recruited and retained to run them. Incentives for participation by industry must be effective, and the structure of these incentives will vary depending on the threat and whether there are markets beyond the US government for the products being sought.

➢ **The United States must have a healthcare system that can more efficiently surge to provide care for the ill and injured during epidemics and catastrophes.**

While our country's preparedness programs have successfully readied our healthcare system to respond well during community-level events, we still have a way to go to truly build the healthcare system's capacity to manage highly contagious patients during an epidemic and to manage patients following major disasters. In the years since 9/11, there have been a range of important efforts to improve the capacity of hospitals to respond to disasters, including the ASPR Hospital Preparedness Program, the National Disaster Medical System, CDC's Public Health Emergency Preparedness Program, and the Medical Reserve Corps. Taken together, these programs have helped to create a cadre of professionals dedicated to healthcare system preparedness for disasters. What used to be largely an after-hours volunteer-only avocation is now a serious preparedness community. As a result of this and of equipment, technology, and infrastructure improvements, many hospitals now have built the capacity to deal with disasters in their communities, and the programs have been valuable in a range of natural disasters.

However, there are important challenges. There is limited capacity to manage highly contagious patients. The response to Ebola cases showed how much advanced training and dedicated staff matter to that kind of response. Specialized infectious disease hospitals have been designated in the aftermath of Ebola, but they will need continued support to maintain that capability. There are also limits to the number of medical or burn casualties that can be cared for in a city or a region. The number varies around the country, but because of financial constraints on hospitals, most preparedness efforts are intended to be able to cope with only small disasters (eg, crashes, building fires, storms) and would not be capable of handling larger epidemics or catastrophes.

Many components of the healthcare system outside of hospitals would be critical to responding to larger events, but they have typically not been included or supported in this work. More of the community will be needed to be engaged to make progress in this work. A new CMS rule may also help expand the system of preparedness beyond hospitals themselves, but it is not clear where the funding will come from to do the work. US government funding for healthcare system preparedness has been cut by more than half from 2002 levels for unclear reasons, stymieing progress. In order to have a healthcare system that can handle a larger number of highly contagious patients or mass medical casualties, we need to build and fund these kinds of efforts.

➢ **The United States needs a public health system that has the expertise and technology to give us earlier warning of outbreaks and disasters and can lead our efforts to deal with them.**

In order to serve as America's first line of health defense against outbreaks, disasters, or the use of biological, chemical, or nuclear weapons, the US public health system is in critical need of 3 basic things: more people, a better funding structure, and modern technology. Unlike the very challenging objective of developing a new medical countermeasure within 6 months of a novel outbreak, it would be fairly straightforward to hire more people and invest in modern technology for our public health departments. But the reduction of funding for public health over the past several years has put such basic requirements out of reach and has weakened our ability to get out in front of outbreaks to limit their impact. The National Health Security Preparedness Index assesses state readiness over a range of capacities, and the most recent scores showed an average state ranking of 6.7 out of 10, a grade that shows that, while there is capacity in the system, there is a long way to go.

For any epidemic or large event involving health consequences, public health leaders and the systems and organizations they lead are a major component of the response. A professional public health preparedness community has been built in the past 15 years, led nationally by the CDC and led locally by state and local health departments. Public health agencies provide top-level scientific analysis and expertise on infectious diseases and epidemics; surveillance programs for early warning of new outbreaks; laboratories for diagnosis and research and the safety programs that go with them; public communication; networks to keep health officials informed; medicine stockpiles; and programs that help build response capacity in communities. But there has been downward funding pressure on the system: Health departments have lost substantial federal resources over the years, and along with that many people. The responses to Ebola and Zika were intensive and resource-draining for many. We seem to fund public health only after an emergency happens, and even then the funding is tied specifically to the emergency at hand, thus restricting the ability of public health departments to build broader capacity over time.

On the other hand, some good news for the country is that there are high numbers of young people pursuing undergraduate and graduate careers in public health. We need more programs and resources to get this rising talent into places where they are needed most. We also should make sure public health has current technologies at its fingertips. Public health particularly needs both more advanced diagnostics and functional IT systems that can connect it to medical providers. In many places, public health lags far behind the private sector in technology investment because budgets have not allowed for it. It will also be important for us to systematically study and learn from our increasingly frequent response to outbreaks nationally and internationally. Not only do we need scientific research to understand the pathogens and diseases, but we need the systems analysis and operations research to study and understand whether our organizations and approaches are working and the extent to which they will scale in more serious biological crises in the future.

➢ **The United States should bolster international efforts to protect the nation by stopping infectious disease at its source and working to prevent bio, chem, and nuclear proliferation.**

One clear lesson from epidemic response in the past 15 years has been that we need to work internationally to help contain outbreaks that develop elsewhere. Not only is it the right thing to do because we have the expertise that can help, it is often the only thing that will prevent the spread of problems to the United States. The International Health Regulations (IHR) have been of great benefit because they have made it an international norm and expectation that countries will prepare themselves to discover and respond to outbreaks, but most countries are not able to meet the IHR standards without substantial technical or other resource support. We all witnessed the complexity and fragility of the international response to Ebola. The WHO reforms for epidemic response coming out of this crisis are therefore of high-stakes importance. So too are the steps that countries take on their own to prepare to help with emergency international epidemic response. In the United States, for example, we need a stronger and faster way to deploy doctors and nurses to help prevent the medical response from collapsing into chaos in extraordinary epidemics.

A development that has been transforming international preparations for globally important epidemics has been the Global Health Security Agenda, a strategy and program that enlists the talent, resources, and commitment of a large group of nations that have resolved to help build epidemic response in countries which are not prepared yet to identify or stop new outbreaks. Recently, countries have undergone first-ever external evaluations of their preparedness efforts, with the hope that these efforts will spur more change, bring in expertise where needed, and shine an international light on the need for national health security and preparedness.

The United States should continue to support and help build the GHSA, because it is in the strong interest of US health and national security to ensure that other countries have the capacity to stop outbreaks before they spread internationally. Since the start of the GHSA, countries are now sharing crucial epidemic planning and response information with each other through transparent external evaluations for the first time. In addition, the GHSA shares the burden for helping to improve countries’ epidemic response capacities, distributing it broadly among nations. An important additional step would be to establish an international index of capacity that would help independently assess progress over time.

In the realm of deliberate biological threats, the BWC continues to be a crucial norm against the development or use of biological weapons. Especially in a time when countries have violated the chemical weapons norm, maintaining the norm against biological weapons is critical. Efforts to maintain or restore global norms against development and use of chemical and nuclear weapons are also now vitally important.

➢ **The United States needs to anticipate and apply powerful new biotechnologies that are coming on-line in order to make the most of them and to manage consequences should they go wrong.**

The United States should maintain its international position and leadership in the biological sciences, both because of the many associated scientific and economic benefits but also because of the need to help shape the emerging ethical, safety, and security rules of the road. It would be hard to miss the biotechnology revolution going on around us. Biotech is a powerful global engine for science, medicine, agriculture, and the economy writ large. CRISPR/Cas is only one of the most visible tools emerging—experts in bioengineering say we should expect transformative tools like it to emerge again and again, suddenly. Countries around the world are making big investments. The gene editing/genome engineering market alone will be worth $5.5 billion in 2021.

The US government should quite deliberately and strategically be scanning the horizon for new technologies and industrial approaches that we can use to help prevent and prepare ourselves for biological threats of the future. Changes to automation, industrialization, informatics, engineering, manufacturing, and more will bring incredible opportunities to transform the way we deal with biological threats.

We also need to anticipate and react wisely to the new challenges, and even dangers, that biotechnology will bring. One example of the kind of challenge that the United States—and the international community—need to address is how to manage research that can result in the creation of novel, highly lethal, highly transmissible viruses that do not exist in nature. The concern is that such work increases the chances that a laboratory accident or security breach could result in exposure of laboratorians or the public to a novel and highly pathogenic virus. This is an issue that became public over the past few years and has resulted in a moratorium on such work. In our view, there is now visible a circumscribed area of experimental work that poses extraordinary pandemic dangers and so should require extraordinary justification if it is to be pursued. The US government is now working through its approach to and policy on these experiments, and the new Administration should attend to this process carefully. Other unexpected developments in biological science will continue to occur and require the attention of the US and other governments. Creating biosafety norms internationally for experiments that have the potential to cause great harm following accidental or deliberate spread should be an international science priority.

➢ **The United States must provide the leadership and organization to advance health security.**

Taken together, the recommendations here and in the commentaries that follow are really about building a more robust health security infrastructure in the United States. This work will immeasurably help the United States to stay ahead of the preparedness curve and to minimize the economic, human health, and societal impacts of outbreaks and disasters that will surely challenge us in the future. Given how broadly distributed health security responsibilities are in the US government, a senior White House leader and staff should be given responsibility for top-level leadership of health security efforts across the government.

Beyond the White House, there need to be leaders in the federal agencies and Congress who are concerned about these challenges, highly informed about developments in infectious diseases and bioscience, and committed to addressing them. Our Center's Emerging Leaders in Biosecurity Fellowship program is intended, in part, to help foster the generation of new leaders who will rise into these positions. Other efforts will be vital to build, educate, and empower this community nationally and internationally, since so much of this work has elements that cross borders.

With strong commitment, focus, and resources, the goals we describe above are within reach. We look forward to working with Congress, the new Administration, and other nongovernment partners to help catalyze and support this vital work.

